# The Semantic Associative Ability in Preschoolers with Different Language Onset Time

**DOI:** 10.3389/fpsyg.2016.01025

**Published:** 2016-07-12

**Authors:** Dina Di Giacomo, Jessica Ranieri, Eliana Donatucci, Nicoletta Caputi, Domenico Passafiume

**Affiliations:** Department of Life, Health and Environmental Sciences, University of L’AquilaL’Aquila, Italy

**Keywords:** semantic associative ability, visuoperceptive semantic, early language, delayed language, typical language

## Abstract

Aim of the study is to verify the semantic associative abilities in children with different language onset times: early, typical, and delayed talkers. The study was conducted on the sample of 74 preschool children who performed a Perceptual Associative Task, in order to evaluate the ability to link concepts by four associative strategies (function, part/whole, contiguity, and superordinate strategies). The results evidenced that the children with delayed language onset performed significantly better than the children with early language production. No difference was found between typical and delayed language groups. Our results showed that the children with early language onset presented weakness in the flexibility of elaboration of the concepts. The typical and delayed language onset groups overlapped performance in the associative abilities. The time of language onset appeared to be a predictive factor in the use of semantic associative strategies; the early talkers might present a slow pattern of conceptual processing, whereas the typical and late talkers may have protective factors.

## Introduction

In the early age, children acquire the concepts observing the context and are able to organize efficiently and functionally their knowledge: progressively, the concepts develop and the semantic store emerges by using of different associative strategies. The use, recall, and functional organization of the concepts in the semantic store represent the basis of semantic competence. In that mechanism, the language represents an important cognitive factor: linguistic and conceptual development converge together in the process of early words learning (Arunachalam and Waxman, 2010). The developmental progression of knowledge is based on features of concepts: the children start from perceptual categorization to arrive to abstract categorization in order to structure the semantic store. In this process, the language represents an important increasing factor of semantic system in childhood. The language appearance in early infancy and its development represents improvement of knowledge competence (Bloom, 2000; Mandler, 2000; Booth et al., 2006; Fulkerson and Waxman, 2007; Waxman and Gelman, 2009). Arunachalam and Waxman (2010) designed mappings about the infant sensitivity to relations between words and concepts: within first year, children set words to commonalitites among objects; in second year, they define precise mappings between kinds of concepts (i.e., categories of objects, properties of objects, relations among objects). Afterward, different traiettories of mappings develop: in first time mapping of nouns emerge and then the mapping for adjectives and verbs. That is due to the different informational requirement for them.

An interesting investigation is analyzing the effect of language onset time like an advatange and/or a disvantage factor in the semantic development. Children’s language emerges typically in range 12–24 months of age, but some children present a variability in terms of to begin talking: some children speak before that time and are called early talkers, whereas some else after that timing and are named late talkers.

Several studies have been conducted on the different language onset time having as focus the expressive language, the morphology and sintax, as that they represent the weaker language endowment (Rescorla, 1989, 2009; Rice et al., 2008; Rescorla and Turner, 2015). Most relevant researches have been conducted on the late talker profile identifying him as child at 2–3 years with delayed vocabulary and sintax but not significant neurological, sensory, or cognitive deficits (Desmarais et al., 2008). Moreover, Rescorla (2013) highlighted like some late talkers have expressive language delay only, whereas others have delayed receptive language.

By contrast the linguistic involvement, few studies have been focused on the effect of language onset time on the semantic competence, in particular on the use of semantic strategies basilar for the knowledge processing.

Previously, our research group investigated the semantic associative using in developmental age showing the first step of semantic processing in terms of associative strategies’ using. Our findings highlighted that beginning at 4 years old, children were able to use the semantic associative relations but that competence increased during cognitive development. In particular, the ability to associate concepts using different strategies has been showed being active since the preschool age. Our research evidenced the progression of semantic associations and the roles they have in the semantic store buinding (Di Giacomo et al., 2012). Perceptual and then linguistic processes co-occur to develop semantic abilities. The child becomes semantically competent during preschool and early school development using sequentially perceptual and verbal encoding (Murphy, 2002; Needham et al., 2006; Coley, 2007; Nguyen, 2007; Di Giacomo et al., 2010, 2012; Herrmann et al., 2012).

Lately, we oriented our focus on the observation of the semantic strategies and the relation with the early or delayed language onset time; we have been interested to evaluate if semantic competence develops independently of language onset time, and finally, if children with early or delayed language acquisition develop semantic ability at different times; to our knowledge, few researchers have focused their interest on this topic.

Overall aim of the present study is to verify the semantic associative abilities in a preschool population tailored for different language onset time (early, delayed, and typical). We wanted to analyze if linguage expressive could be related to the flexibility of conceptual processing.

The study was conducted on a preschool sample from a population with language development in progress, and we assumed that the increase in linguistic competence from 3 to 6 years of age would provide a better analysis of the possible influence of language on conceptual development by visuoperceptual elaboration.

## Materials and Methods

### Subjects

The participants are 74 preschool children (39 female and 35 male) with mean age 4.1 years (*SD* = 0.8) distributed in three groups defined by phase of language onset: (i) the Early Language (EL) group included 17 children with mean age 3.9 (*SD* = 0.8) with early language onset (Mean = 7.8 months and *SD* = 0.5); (ii) the Typical Language Language (TL) group included 39 with mean age 4.4 (*SD* = 0.8) and with typical language onset (mean = 11.3 months and *SD* = 1.2); (iii) the Delayed Language group (DL) included 18 children with mean age 3.8 (*SD* = 0.7) with delayed language onset (mean = 17.3 months and *SD* = 2.9). The distribution of the sample in the three groups was made on the basis of pediatric evaluations, parents’ reports on the basis of Rescorla’s criteria (Rescorla, 1989): age of acquisition of first words, age of gesture indication, and age of spontaneous use of first phrases (**Table 1**).

**Table 1:** Demoghaphic data of the participants.

Variables	Sample			*F*	*p*
			
	Early language	Tyrpical language	Delayed language		
N° (Total *n* = 74)	17	39	18		
Male	10	14	11		
Female	7	25	7		
Age	3.9 (±0.8)	4.3 (±0.7)	3.8 (±0.7)		
Age mother	37.9 (±5.6)	36.7 (±5.5)	39.7 (±5.4)		
Age father	40.8 (±5.4)	40.5 (±6.3)	42.3 (±2.6)		
Gesture age	7.5 (±1.15)	8.2 (±1.7)	12.1 (±3.2)	32.4	0.00^∗^
First word age	9.2 (±2.5)	10.9 (±1.9)	15.6 (±4.2)	18.2	0.00^∗^
Raven test	14.2 (±4.4)	17.2 (±4.9)	14.2 (±5.3)	2.4	0.09
*t* Raven test	5.4 (±3.7)	4.8 (±1.7)	3.4 (±1.5)	2.6	0.08


*^∗^statistically significant.*

Excluded children have been n. 74 because their performance have been under theresold by Raven test (see Test).

All children lived with both parents.

### Test

A standardized psychological battery was administered.

*Raven’s Colored Progressive Matrices* (Italian Adaptation Belacchi et al., 2008) is a non-verbal test widely applied in the evaluation of general intelligence, and is composed of 36 items. The subject was asked to choose from a set of six, the piece that was missing in a target pattern. The standard score was analyzed. The Raven’s Colored Progressive Matrices was used to measure the cognitive competence of the subjects in order to exclude those with cognitive deficits/difficults.

*Prova di Associazione Semantica (PAS*, Semantic Associative Task, Di Giacomo and Passafiume, 2014) is a visuoperceptual task to evaluate the semantic associative abilities. It was carried out on native Italian speaking children. The task was composed of two sets: Naming and Matching tasks.

#### Naming Task

**FIGURE 1:**
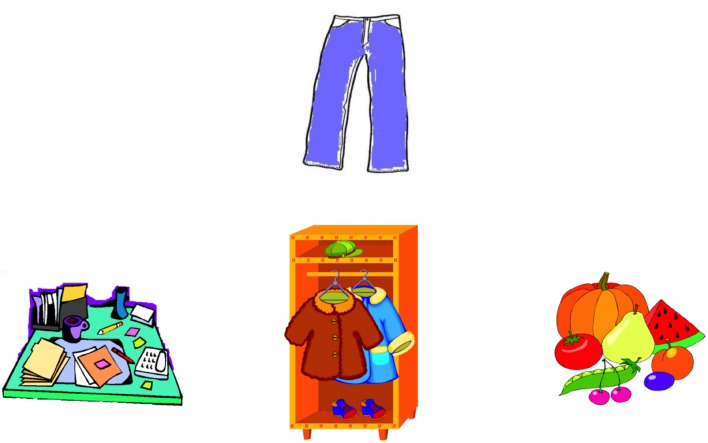
**Example of Matching item**.

The Naming task consists of 40 drawing items representing objects applied in the Matching task. The examinator asks the subject to say the name of the drawn object (**Figure 1**). The Naming task is a preliminary test to measure the children’s ability to recognize the targets used in the Matching task (cut-off is 75% correct respnses). The score is the sum of correct responses.

#### Matching task

**FIGURE 2:**
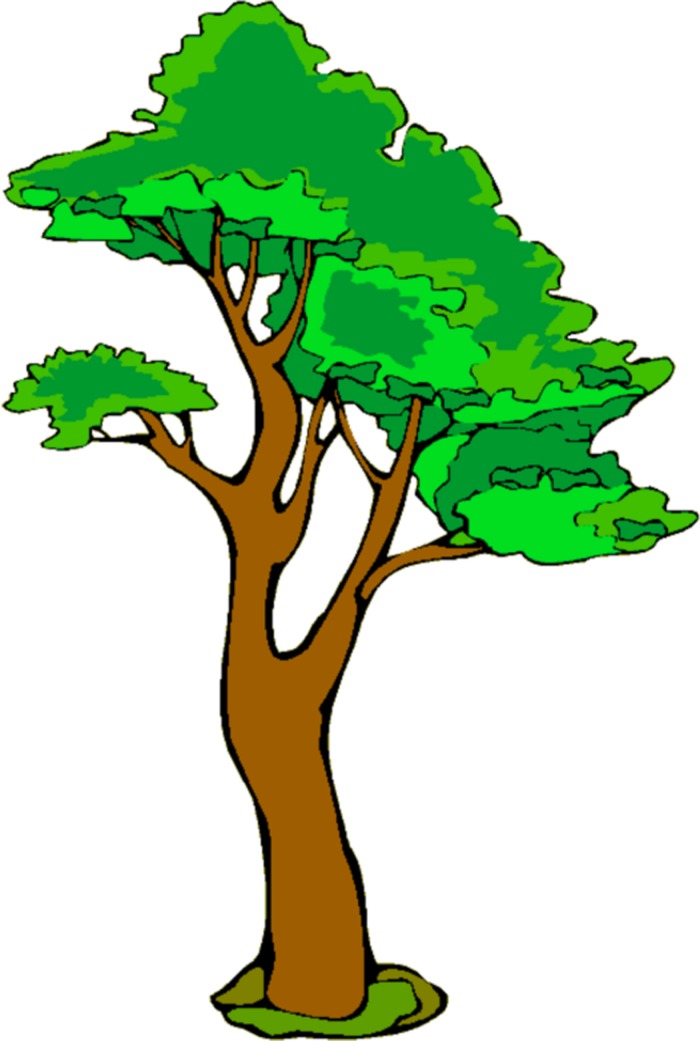
**Example of Naming item**.

Matching task is composed of 40 items and each item includes one target object [and three other objects (see **Figure 2**)]. The examinator asks to the subject to indicate which one of the three choises (objects) is related better than others to the target. The items investigate four semantic associative relations: (i) Function, (ii) Part/Whole, (iii) Contiguous, and (iv) Superordinate). The associative relations were as follows: the Function category consists of pairing an object with its use (e.g., scissors and to cut); the Part/Whole category consists of pairing an object with its single part (e.g., fish and fin); the Contiguous category consists of pairing an object with its complement (e.g.,. pencil and eraser); the Superordinate category consists of pairing an object with its class membership (e.g., dog and animal). Three trial items applied. The score was the sum of the correct responses. The Cronbach α value is: function = 0.83; part/whole = 0.86; contiguity = 0.80; superordinate = 0.80).

In addition, the time was measured for the subject’s completion of the Naming and Matching tasks.

### Procedure

The children have been recruited in pediatric ambulatory and kindergarten school. The children have been evaluated by Psychologists in individual sessions lasting 45 min in a quiet and dedicated room. The scoring of psychological tests was get by judges were blind by the study’s objectives. Parents have been proposed a individual interview lasted at least 1 h in order to have more information about linguistic ability of their children. Written informed consensus by parents was mandatory and obtaneid.

Data was inserted in the Case Report form builded for this research.

### Ethic Statement

The study was carried out with the Positive Opinion of Ethic Commetee of University of L’Aquila (Italy).

### Plan Statistical Analysis

The data were submitted to statistical analysis with value α < 0.05. The statistical analysis were performed through the Statistica software.

Descriptive statistics (mean and standard deviation for numeric variables, frequencies, frequencies for categorical variables) were processed for all variables examined.

An ANOVA analysis was applied to match the semantic performance difference in three groups (TL, EL, and DL), and then we conducted the *post hoc* analysis (Tukey test). Suddenly, we conducted MANOVA to compare the age groups and the language onset time groups to evaluate the effect of aging and the language onset time on the semantic performance. The aging effect is expected.

## Results

Aim of the research was to analyze the semantic associative performance in early developmental age. Our focus has been the use of associative strategies in the range age 3–6 years old, in a tailored sample by different language onset time.

First, we wanted to analyze the influence of age in the elaboration of semantic associative strategies. The sample has been divided in three groups by the chronological age: (i) 3-year-old group was composed of 21 subjects, (ii) 4-year-old group was composed of 26 subjects, and (c) 5-year-old group was composed of 27 subjects. In **Table 2**, we reported raw score.

**Table 2:** Raw scores of age groups in the experimental tasks.

Tests	Age groups		
	
	3-years-old	4-years-old	5-years-old
Naming	29.6 (±7.0)	34.4 (± 3.7)	34.9 (±3.2)
Matching	30.5 (±5.8)	37.9 (± 1.1)	34.4 (±6.9)
Function	7.9 (±1.5)	9.1 (±0.8)	9.6 (±0.6)
Part/Whole	7.2 (±1.5)	8.9 (±1.5)	9.0 (±0.8)
Contiguity	7.9 (±1.4)	9.0 (±0.9)	9.6 (±0.6)
Superordinate	7.3 (±2.3)	9.2 (±0.9)	9.6 (±0.7)


**FIGURE 3:**
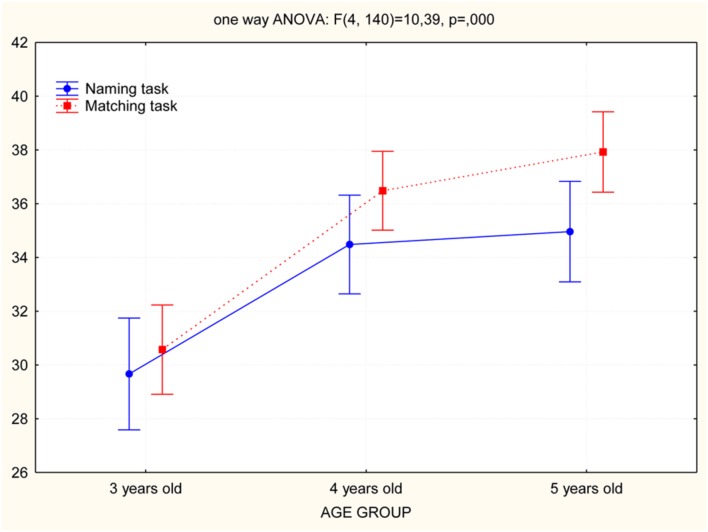
**Comparison of age groups performance in Naming and Matching tasks**.

A MANOVA 3 (age groups) × 2 (tasks: Naming, Matching) evidenced significant difference among the three groups in the two tasks [Naming: *F*(2,71) = 8.4; *p* = 0.001, and η^2^ = 0.19; Matching: *F*(2,71) = 23.5; *p* < 0.0001, and η^2^ = 0.39]. The *Post hoc* analysis (Tukey test) showed that in the Naming task, the 3-year-old group was significantly different from the 4-year-old (*p* < 0.002), and 5-year-old groups (*p* < 0.001) while no significative difference were found between the 4- and 5-year-old groups. Significant differences were also found in the Matching task: the 3-year-old group was less able than the 4-year-old (*p* < 0.001) and 5-year-old groups (*p* < 0.004; **Figure 3**). The expected results have confirmed out the previous data (Di Giacomo et al., 2012).

**FIGURE 4:**
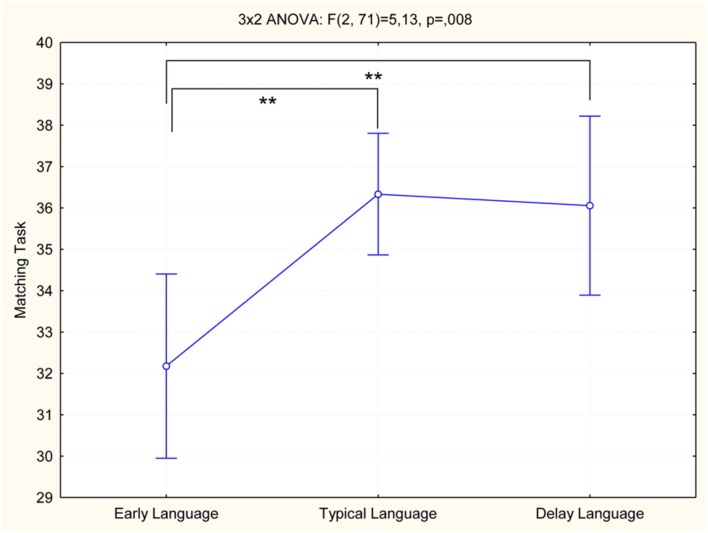
**Representation of the perfromance of EL, TL, and DL groups in Matching task.** ^∗∗^statistically significant.

Then, we have conducted a statistical analysis to evaluate the performance of the three language onset time groups (EL, TL, and DL) in the associative test (Naming and Matching task). **Table 3** reported the raw score of the sample distributed in language onset time. A 3 × 2 MANOVA showed differences between language onset time groups in semantic tasks [*F*(4,140) = 2.94; *p* = 0.02, and η^2^ = 0.78]. The *Post hoc* analysis (Tukey test) evidenced different performance between language onset groups only in the Matching task: the EL group’s scores were lower than TL (*p* < 0.001) and DL (*p* < 0.004) groups (**Figure 4**).

**Table 3:** Raw scores of language onset time groups in the experimental tasks

Tests	Language onset time groups		
	
	Early language	Typical language	Delayed language
Naming	31.9 (±3.9)	34.4 (±4.5)	33.7 (±6.3)
Matching	32.5 (±5.7)	36.0 (±4.7)	34.4 (±6.9)
Function	8.3 (±1.3)	9.2 (±1.1)	8.7 (±1.7)
Part/Whole	7.5 (±1.8)	8.7 (±1.2)	8.6 (±1.5)
Contiguity	8.3 (±1.2)	9.0 (±1.3)	8.5 (±2.2)
Superordinate	8.2 (±2.1)	8.9 (±1.7)	8.4 (±2.2)


**FIGURE 5:**
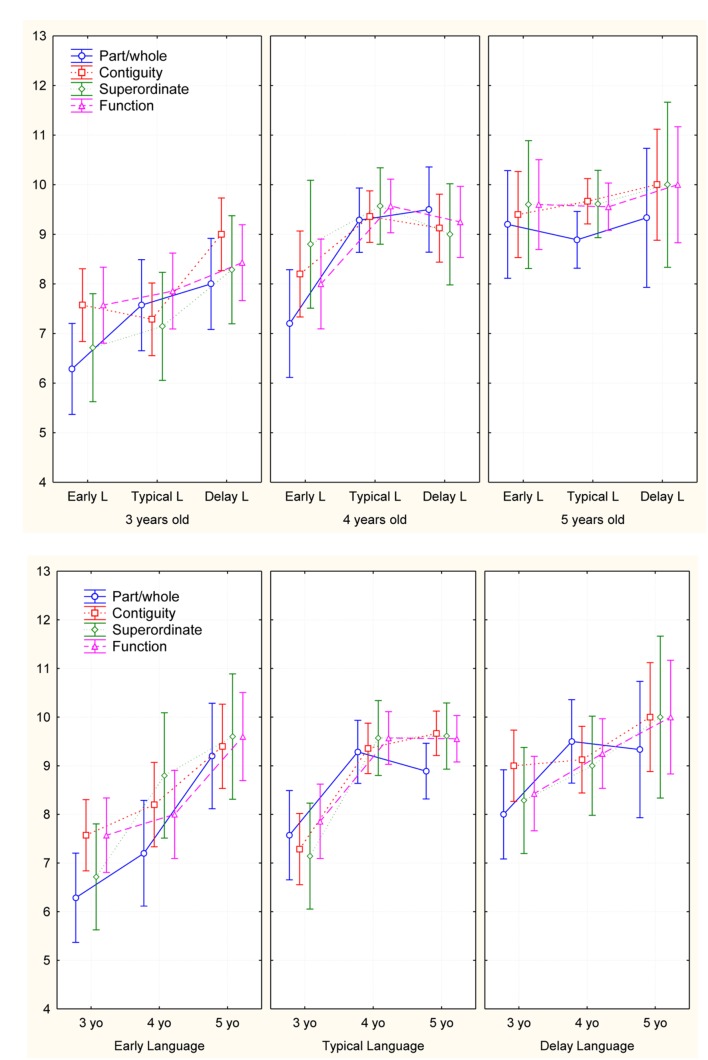
**Representation of the Matching task performance by age and language onset groups**.

Besides, a 3 (language onset time groups) × 3 (age groups) × 4 (types of semantic associations: function, part/whole, contiguity, and superordinate) MANOVA showed a significant difference among the age groups [*F*(8,124) = 2.3; *p* < 0.001, and η^2^ = 0.25] and the onset language groups [*F*(8,124) = 5.34; *p* < 0.02, and η^2^ = 0.13], but no significant interaction between age and language onset time groups. This result is interesting: the aging effect isn’t affect the semantic associative performance of children with different language onset time (**Figure 5**).

**FIGURE 6:**
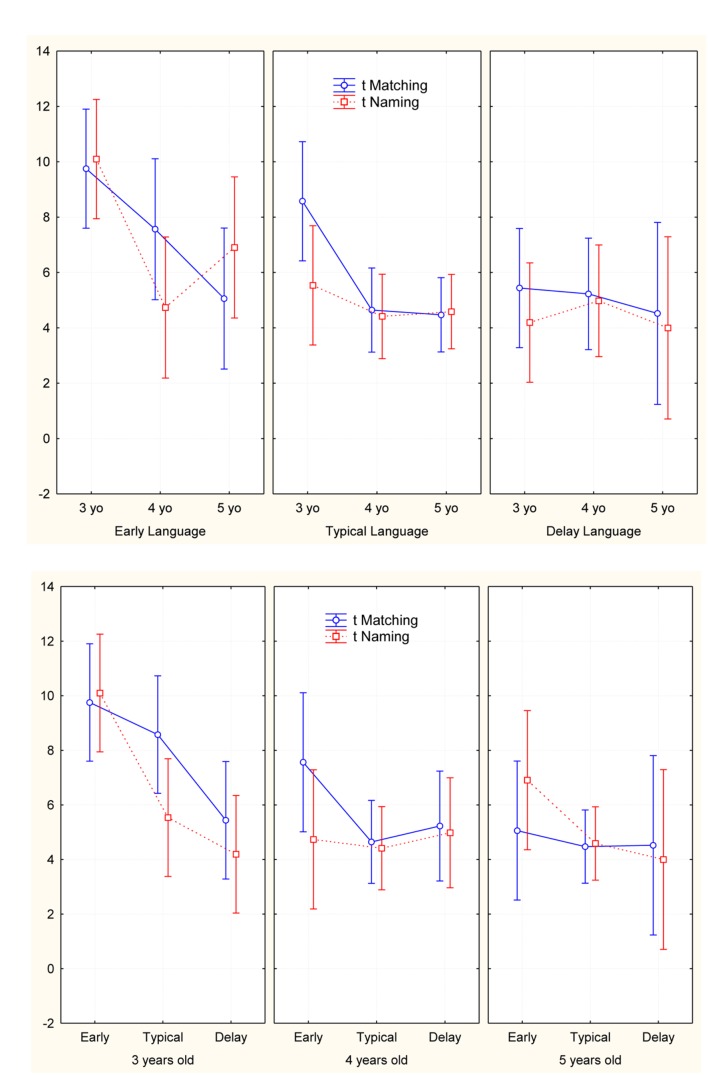
**Representation of time execution in Naming and Matching tasks**.

Finally, we have analyzed the execution time (*t*) of sample in Naming and Matching taskes. A 3 (EL, TL, and DL groups) × 3 (age groups) × 2 (*t* Naming and Matching tasks) MANOVA evidenced significant differences in language onset time groups [*F*(4,128) = 2.7; *p* < 0.03, and η^2^ = 0.78] and age groups [*F*(4,128) = 3.4; *p* < 0.01, and η^2^ = 0.09]; Tukey test showed in EL performance resulting slower than TL and DL groups in Matching task (*p* < 0.001); TL and DL groups performance appear similar. The *post hoc* on age groups performance evidenced the older children (4- and 5-year-olds) faster than younger (3-year-olds) (*t* Naming: *p* < 0.05, *t* Matching: *p* < 0.008) (**Figure 6**).

## Discussion and Conclusion

The present study proposed to analyze the impact of the language onset time in the development of associative strategies using. Particularly, we wanted to verify if the semantic ability in early childhood could be affected by language onset time, reflecting specific features as well as linguistic competence.

Our data showed that language onset time does not seem to affect directly the use of examined semantic associative abilities. The children improve their using of associative strategies during cognitive development, without significant linkage to verbal production. The data evidenced that the children with delayed language are able to use the associative strategies as well as the children with typical language: these performance appear in the elaboration of information and in execution time of semantic task.

Our findings showed developing semantic ability isn’t related primarly to the language onset time. The performance of Delayed Language Onset Group didn’t be different from the Typical Language Onset Group on the Matching Task; morevor, DL performance have differed from EL in both measurements (correctness and execution time). The early language children have been less efficient than the other subjects of two groups in the concepts association and the use of single associative relations. The early language group appeared weak in the use of contiguity and part/whole relations.

Our results suggest that semantic association competence and the age of linguistic production aren’t directly linked, even though the early word production could predict a weakness in the managing of the linkage of the concepts; in contrary, the delayed linguistic production didn’t seem to influence the development of associative strategies.

Several studies demonstrated the delayed lexical activation could reflect a weakness in language development and favoring bloomer and/or late talker outcomes (Rescorla, 2005; Rice et al., 2008). Rice et al. (2008) conducted a follow up study of the evolution of the performance of late talking children at 3-year-old: the research demonstrated the persistence of linguistic impairment connected to the syntactic and grammatical deficit and a relative deficit in the semantic quotient (verbal task) with important involvement of spontaneous language.

Rescorla (2009) showed that the linguistic difficulties persisted into adolescence. Follow-up studies evidenced such as the language that initially have evolution difficulties during the develop maintain critical even if supported linguistic rehabilitation interventions. These conclusions are supported by several reports (Rescorla, 2005; Rice et al., 2008). Our research highlighted the importance of the strengh of conceptual flexibility in subjects with delayed language onset.

Few studies have focused on the early talkers. Our results suggested that the early talkers have a weakness in their semantic competence: though their verbal production is early, their development of conceptual associative strategies is later than typical and delayed talkers. They performed well in the Naming task, but not in the Matching Task. We added to a development model of the semantic and conceptual stores the finding that late talkers demonstrate stronger conceptual processing. Several studies investigated the grammatical and lexical difficulties in cognitive development; in our research the late talkers showed more competence in the use of semantic associative strategies. Furthermore, early talking can be considerate a predictive factor for use in educational systems to improve semantic ability since to early time. In the applied psychology, and in particular in the educational stimulation, our results can contribute to the formulation of interventation planning more efficiently focused on integration of the competences of the conceptual and semantic memory on child with delayed onset language. Semantic categorization can be used as a competence on which building, through use of plans to stimulate and promote linguistic performance. The meanings of the words and the linkages between them might improve the outcomes of the educational stimulation, and later, increase verbal production.

## Author Contributions

All authors listed, have made substantial, direct and intellectual contribution to the work, and approved it for publication.

## Conflict of Interest Statement

The authors declare that the research was conducted in the absence of any commercial or financial relationships that could be construed as a potential conflict of interest.
